# Poly[diaqua­(μ_4_-carboxyl­atomethyl­phospho­nato)(μ_4_-carb­oxy­methyl­phospho­nato)penta­deca­methyl­penta­tin(IV)]

**DOI:** 10.1107/S1600536813000676

**Published:** 2013-01-19

**Authors:** Mouhamadou Sembene Boye, Aminata Diasse-Sarr, Arnaud Grosjean, Philippe Guionneau

**Affiliations:** a Departement de Chimie, Faculte des Sciences et Techniques, Universite Cheikh Anta, Diop, Dakar, Senegal; b CNRS, Univ. Bordeaux, ICMCB, UPR 9048, 87 avenue du Dr A. Schweitzer, F-33608 Pessac, France

## Abstract

The central Sn^IV^ atom of the penta­nuclear title complex, {[Sn(CH_3_)_3_]_3_O_2_C(CH_2_)PO_3_[Sn(CH_3_)_3_(H_2_O)]_2_HO_2_C(CH_2_)PO_3_}, is located on a twofold rotation axis; due to symmetry, the H atom of the carboxyl group of the anion is disordered with a site occupancy of 0.5. The central Sn^IV^ atom is bonded to three methyl groups (one of which is disordered about the twofold rotation axis) and is symmetrically *trans* coordinated by two phospho­nate groups with Sn—O = 2.2665 (12) Å while the other SnMe_3_ residues are asymmetrically *trans* coordinated with Sn—O = 2.1587 (12) and 2.3756 (13) Å for one residue and Sn—O = 2.1522 (12) and 2.4335 (12) Å for the other; the Sn–O distances involving two O atoms *trans* to carboxyl­ate are longer than those *trans* to phospho­nate groups. The Sn—C distances lie in a very narrow range [2.112 (2)–2.133 (3) Å]. The oxyanion behaves as a tetra-coordinating ligand. The bridging mode of the latter leads to the formation of layers parallel to (001) that are inter­connected by O—H⋯O and C—H⋯O hydrogen bonds.

## Related literature

For applications of tin-based materials, see: Dutrecq *et al.* (1992 ▶); Basu Baul *et al.* (2011 ▶). For related structures, see: Zhang *et al.* (2010 ▶).
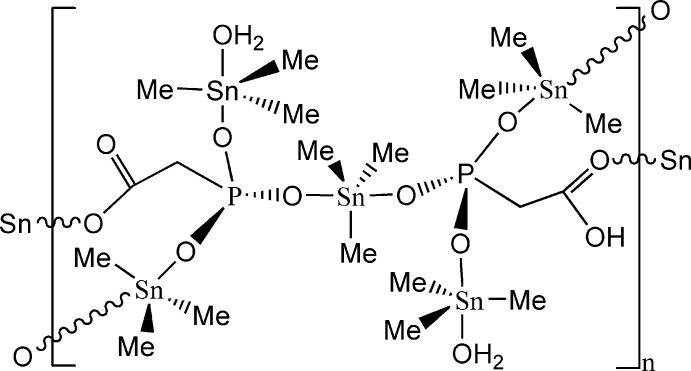


## Experimental

### 

#### Crystal data

[Sn_5_(CH_3_)_15_(C_2_H_2_O_5_P)(C_2_H_3_O_5_P)(H_2_O)_2_]
*M* *_r_* = 1130.01
Monoclinic, 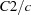

*a* = 11.6939 (2) Å
*b* = 13.1689 (3) Å
*c* = 25.9575 (5) Å
β = 95.40 (1)°
*V* = 3979.61 (14) Å^3^
*Z* = 4
Mo *K*α radiation
μ = 3.22 mm^−1^
*T* = 150 K
0.32 × 0.15 × 0.15 mm

#### Data collection

Nonius KappaCCD diffractometer
Absorption correction: multi-scan (*SCALEPACK*; Otwinowski & Minor, 1997 ▶) *T*_min_ = 0.426, *T*_max_ = 0.644
15302 measured reflections
7932 independent reflections
7236 reflections with *I* > 2σ(*I*)
*R*_int_ = 0.020

#### Refinement

*R*[*F* ^2^ > 2σ(*F* ^2^)] = 0.022
*wR*(*F* ^2^) = 0.054
*S* = 1.09
7932 reflections
182 parameters
H-atom parameters constrained
Δρ_max_ = 1.14 e Å^−3^
Δρ_min_ = −0.85 e Å^−3^

### 

Data collection: *COLLECT* (Nonius, 2003 ▶); cell refinement: *SCALEPACK* (Otwinowski & Minor, 1997 ▶); data reduction: *DENZO* (Otwinowski & Minor, 1997 ▶); program(s) used to solve structure: *SHELXS97* (Sheldrick, 2008 ▶); program(s) used to refine structure: *SHELXL97* (Sheldrick, 2008 ▶); molecular graphics: *ORTEP-3* (Farrugia, 2012 ▶); software used to prepare material for publication: *publCIF* (Westrip, 2010 ▶).

## Supplementary Material

Crystal structure: contains datablock(s) I, global. DOI: 10.1107/S1600536813000676/pv2611sup1.cif

Structure factors: contains datablock(s) I. DOI: 10.1107/S1600536813000676/pv2611Isup2.hkl

Additional supplementary materials:  crystallographic information; 3D view; checkCIF report

## Figures and Tables

**Table 1:** Hydrogen-bond geometry (Å, °)

*D*—H⋯*A*	*D*—H	H⋯*A*	*D*⋯*A*	*D*—H⋯*A*
O6—H1*O*⋯O5^i^	0.89	1.83	2.693 (2)	164
O6—H2*O*⋯O1^i^	0.85	1.88	2.706 (2)	161
C9—H9*A*⋯O4^ii^	0.99	2.51	3.227 (2)	129

Symmetry codes: (i) 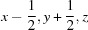; (ii) 
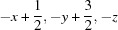
.

**Fig. 1.**
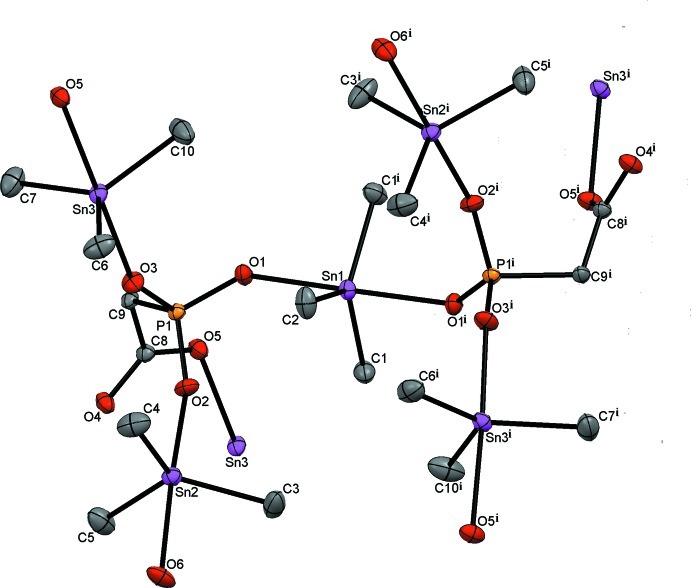
A view of the asymmetric unit of the title compound showing the numbering scheme. Displacement ellipsoids are drawn at the 30% probability level. H-atoms have been omitted for clarity. Symmetry code: i = -x + 1, y, -z + 1/2

**Fig. 2.**
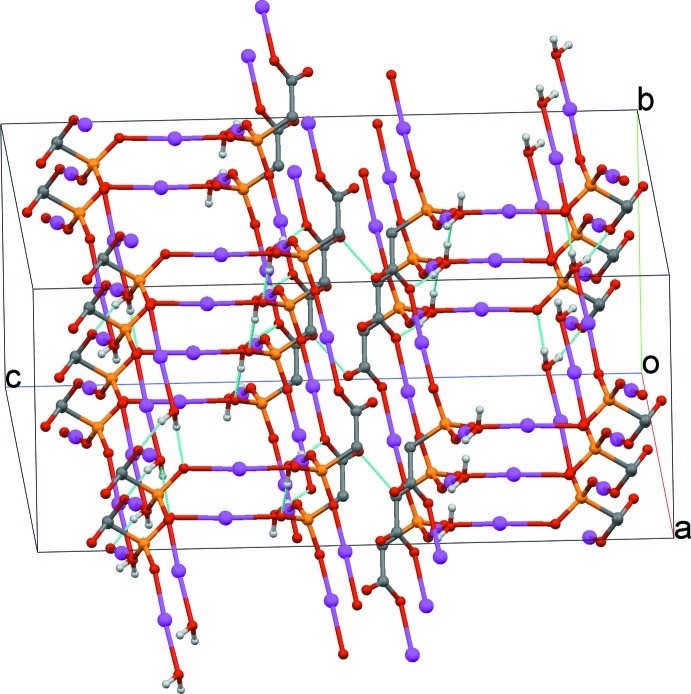
A view of O—H···O hydrogen bonds in the crystal structure. H atoms non-participating in hydrogen bonding and carbon atoms of the Sn- methyl group have been omitted for clarity.

## supplementary crystallographic information

### Comment

Organotin(IV) compounds are involved in many applications in a large diversity of fields including agriculture, medicine and industry (Dutrecq *et al.*, 1992; Basu Baul *et al.*, 2011). Carboxylalkylphosphonate derivatives are interesting from the point of view of their structural tendency to form polymeric structures. The presence of carboxylate and phosphonate functions makes carboxylalkylphosphonate a polyfunctional ligand which can coordinate to the metal in all directions. The aim of combining organotin and carboxyalkylphosphonate is to exalte the biocidal activity in the resulting title complex. Only a few structures related to the title compound have been reported (Zhang *et al.*, 2010).

The asymmetry unit of the title complex, [(SnMe_3_)_3_O_2_C(CH_2_)PO_3_(SnMe_3_.H_2_O)_2_HO_2_C(CH_2_)PO_3_]_n_, contains one half of the trimethyltin(IV) (SnMe_3_) lying on a 2-fold axis linked to a [(SnMe_3_)2.H_2_O](PO_3_CH_2_CO_2_H_0.5_) fragment leading to the pentanuclear complex (Fig. 1) that forms the repeat unit of the polymer; the hydroxyl H-atom is disordered with 0.5 occupancy factor. The geometry of all tin atoms (Sn1, Sn2 and Sn3) is trigonal bipyramidal. The Sn—O distances in the title complex involving two O atoms *trans* to carboxylate [Sn3—O5^ii^ = 2.4335 (12) Å] are longer than those *trans* to phosphonate groups [Sn3—O3 = 2.1522 (12) Å] (Table 1). The longest P—O bond [P1—O3 = 1.5156 (12) Å] is linked to the strongest Sn—O bond while, on the contrary, the shortest C—O bond [C8—O5 = 1.257 (2) Å] is linked to the weakest one. The values of O—Sn—O angles are in the range [176.51 (5)–176.84 (6)°] indicating a significant deviation from linearity. The C—Sn—C angles [115.13 (11)–124.85 (9)°] indicate almost planar Sn—C_3_ groups. The crystal structure obtained is three-dimensional since, within the packing, each pentacoordinated tin atom is bonded to two oxygen atoms in axial positions and three methyl groups in equatorial positions. The SnC_3_ residues are asymmetrically *trans* coordinated. The oxoanion behaves as a tetradentate ligand involving three oxygen atoms of the phosphonate and one of the carboxylate oxygen atom. Resulting chains are interconnected by O—H ···O hydrogen bonds which generate crystal lattice rectangular (Fig. 2 & Table 1).

### Experimental

The title compound was synthesized by the reaction in ethanol (30 ml) of carboxymethylphosphonic acid (0.161 g, 1.13 mmol), KOH (0.229 g, 3.39 mmol) and trimethyltin(IV) chloride (0.675 g, 3.39 mmol) in a 1:3:3 ratio. The mixture was stirred around two hours at room temperature. Suitable crystals for X-ray diffraction were obtained after a slow evaporation of the solvent; m.p. 463–464 K.

### Refinement

Water H atoms were found in a difference map and included at those positions. Other H atoms were placed in geometrically calculated positions with C–H = 0.98 Å for methyl-H and 0.99 Å for methyelene-H, and refined using a riding model with *U*_iso_(H)= *x U*_eq_ (carrier atom); *x* = 1.2 or 1.5.

### Crystal data

**Table:**

[Sn_5_(CH_3_)_15_(C_2_H_2_O_5_P)(C_2_H_3_O_5_P)(H_2_O)_2_]	*F*(000) = 2176
*M**_r_* = 1130.01	*D*_x_ = 1.886 Mg m^−^^3^
Monoclinic, *C*2/*c*	Mo *K*α radiation, λ = 0.71073 Å
Hall symbol: -C 2yc	Cell parameters from 34199 reflections
*a* = 11.6939 (2) Å	θ = 1.0–33.7°
*b* = 13.1689 (3) Å	µ = 3.22 mm^−^^1^
*c* = 25.9575 (5) Å	*T* = 150 K
β = 95.40 (1)°	Prism, colorless
*V* = 3979.61 (14) Å^3^	0.32 × 0.15 × 0.15 mm
*Z* = 4	

### Data collection

**Table:**

Nonius KappaCCD diffractometer	7932 independent reflections
Radiation source: fine-focus sealed tube	7236 reflections with *I* > 2σ(*I*)
Graphite monochromator	*R*_int_ = 0.020
φ and ω scans with κ ofset	θ_max_ = 33.8°, θ_min_ = 2.3°
Absorption correction: multi-scan (*SCALEPACK*; Otwinowski & Minor, 1997)	*h* = −18→18
*T*_min_ = 0.426, *T*_max_ = 0.644	*k* = −20→20
15302 measured reflections	*l* = −40→40

### Refinement

**Table:**

Refinement on *F*^2^	Secondary atom site location: difference Fourier map
Least-squares matrix: full	Hydrogen site location: inferred from neighbouring sites
*R*[*F*^2^ > 2σ(*F*^2^)] = 0.022	H-atom parameters constrained
*wR*(*F*^2^) = 0.054	*w* = 1/[σ^2^(*F*_o_^2^) + (0.0207*P*)^2^ + 4.2723*P*] where *P* = (*F*_o_^2^ + 2*F*_c_^2^)/3
*S* = 1.09	(Δ/σ)_max_ = 0.005
7932 reflections	Δρ_max_ = 1.14 e Å^−^^3^
182 parameters	Δρ_min_ = −0.85 e Å^−^^3^
0 restraints	Extinction correction: *SHELXL97* (Sheldrick, 2008), Fc^*^=kFc[1+0.001xFc^2^λ^3^/sin(2θ)]^-1/4^
Primary atom site location: structure-invariant direct methods	Extinction coefficient: 0.00041 (3)

### Special details

**Table:**

Geometry. All s.u.'s (except the s.u. in the dihedral angle between two l.s. planes) are estimated using the full covariance matrix. The cell s.u.'s are taken into account individually in the estimation of s.u.'s in distances, angles and torsion angles; correlations between s.u.'s in cell parameters are only used when they are defined by crystal symmetry. An approximate (isotropic) treatment of cell s.u.'s is used for estimating s.u.'s involving l.s. planes.
Refinement. Refinement of *F*^2^ against ALL reflections. The weighted *R*-factor *wR* and goodness of fit *S* are based on *F*^2^, conventional *R*-factors *R* are based on *F*, with *F* set to zero for negative *F*^2^. The threshold expression of *F*^2^ > 2σ(*F*^2^) is used only for calculating *R*-factors(gt) *etc*. and is not relevant to the choice of reflections for refinement. *R*-factors based on *F*^2^ are statistically about twice as large as those based on *F*, and *R*- factors based on ALL data will be even larger.

### Fractional atomic coordinates and isotropic or equivalent isotropic displacement parameters (Å^2^)

**Table:**

	*x*	*y*	*z*	*U*_iso_*/*U*_eq_	Occ. (<1)
Sn1	0.5000	0.755553 (11)	0.2500	0.01394 (4)	
Sn3	0.657492 (10)	0.979143 (8)	0.093373 (4)	0.01521 (3)	
Sn2	0.178461 (10)	0.935201 (8)	0.137009 (4)	0.01528 (3)	
P1	0.43748 (3)	0.80781 (3)	0.118293 (15)	0.01189 (7)	
O5	0.33357 (11)	0.57344 (9)	0.08457 (5)	0.0182 (2)	
O4	0.24248 (11)	0.67603 (9)	0.02767 (5)	0.0180 (2)	
O1	0.50422 (11)	0.75081 (9)	0.16291 (5)	0.0161 (2)	
O3	0.49774 (11)	0.90382 (9)	0.10334 (5)	0.0186 (2)	
C7	0.6528 (2)	0.94022 (17)	0.01376 (8)	0.0302 (4)	
H7A	0.6136	0.9940	−0.0072	0.045*	
H7B	0.6112	0.8761	0.0076	0.045*	
H7C	0.7314	0.9326	0.0041	0.045*	
O2	0.31351 (10)	0.82643 (9)	0.12850 (5)	0.0183 (2)	
C9	0.43300 (13)	0.72417 (12)	0.06161 (6)	0.0139 (3)	
H9A	0.4299	0.7663	0.0299	0.017*	
H9B	0.5046	0.6836	0.0634	0.017*	
C2	0.5000	0.9175 (2)	0.2500	0.0258 (5)	
H2A	0.4976	0.9424	0.2143	0.039*	0.50
H2B	0.5699	0.9424	0.2698	0.039*	0.50
H2C	0.4325	0.9424	0.2658	0.039*	0.50
C8	0.33124 (13)	0.65301 (12)	0.05775 (6)	0.0135 (3)	
C1	0.34603 (16)	0.66906 (15)	0.24300 (7)	0.0225 (3)	
H1A	0.3063	0.6788	0.2084	0.034*	
H1B	0.2962	0.6915	0.2691	0.034*	
H1C	0.3645	0.5970	0.2482	0.034*	
C4	0.28441 (19)	1.06554 (15)	0.13493 (10)	0.0303 (4)	
H4A	0.2371	1.1245	0.1239	0.045*	
H4B	0.3420	1.0543	0.1105	0.045*	
H4C	0.3230	1.0781	0.1695	0.045*	
C5	0.07938 (19)	0.89233 (17)	0.06770 (8)	0.0298 (4)	
H5A	0.1051	0.8258	0.0564	0.045*	
H5B	0.0891	0.9429	0.0408	0.045*	
H5C	−0.0018	0.8885	0.0740	0.045*	
C3	0.1385 (2)	0.86901 (18)	0.20725 (8)	0.0326 (5)	
H3A	0.2046	0.8755	0.2332	0.049*	
H3B	0.1204	0.7970	0.2016	0.049*	
H3C	0.0721	0.9038	0.2195	0.049*	
C6	0.59248 (18)	1.11584 (15)	0.12221 (9)	0.0284 (4)	
H6A	0.5612	1.1025	0.1553	0.043*	
H6B	0.5316	1.1424	0.0973	0.043*	
H6C	0.6546	1.1658	0.1274	0.043*	
O6	0.02501 (12)	1.04809 (10)	0.14945 (6)	0.0235 (3)	
C10	0.76318 (18)	0.88614 (18)	0.14468 (9)	0.0339 (5)	
H10A	0.8142	0.9290	0.1675	0.051*	
H10B	0.8093	0.8413	0.1247	0.051*	
H10C	0.7149	0.8451	0.1655	0.051*	
H1O	−0.0374	1.0436	0.1273	0.050*	
H2O	0.0350	1.1118	0.1539	0.050*	
H1O4	0.2273	0.6343	0.0029	0.050*	0.50

### Atomic displacement parameters (Å^2^)

**Table:**

	*U*^11^	*U*^22^	*U*^33^	*U*^12^	*U*^13^	*U*^23^
Sn1	0.01612 (7)	0.01318 (7)	0.01246 (7)	0.000	0.00108 (5)	0.000
Sn3	0.01622 (5)	0.01419 (5)	0.01498 (5)	−0.00358 (3)	0.00010 (4)	0.00139 (3)
Sn2	0.01507 (5)	0.01522 (5)	0.01564 (6)	0.00115 (3)	0.00182 (4)	−0.00054 (4)
P1	0.01177 (16)	0.01130 (16)	0.01267 (17)	−0.00176 (12)	0.00149 (13)	0.00042 (13)
O5	0.0157 (5)	0.0162 (5)	0.0220 (6)	−0.0029 (4)	−0.0018 (5)	0.0060 (4)
O4	0.0163 (5)	0.0181 (5)	0.0183 (5)	−0.0042 (4)	−0.0044 (4)	0.0046 (4)
O1	0.0189 (6)	0.0169 (5)	0.0123 (5)	0.0013 (4)	0.0005 (4)	0.0005 (4)
O3	0.0183 (6)	0.0140 (5)	0.0232 (6)	−0.0066 (4)	0.0010 (5)	0.0020 (4)
C7	0.0352 (11)	0.0341 (11)	0.0217 (9)	−0.0133 (8)	0.0055 (8)	−0.0054 (8)
O2	0.0134 (5)	0.0166 (5)	0.0258 (6)	0.0008 (4)	0.0063 (4)	−0.0011 (5)
C9	0.0130 (6)	0.0166 (7)	0.0123 (6)	−0.0039 (5)	0.0017 (5)	0.0001 (5)
C2	0.0419 (16)	0.0156 (10)	0.0194 (11)	0.000	0.0006 (11)	0.000
C8	0.0130 (6)	0.0135 (6)	0.0140 (6)	−0.0020 (5)	0.0013 (5)	0.0000 (5)
C1	0.0201 (8)	0.0262 (9)	0.0207 (8)	−0.0057 (6)	−0.0012 (6)	0.0045 (7)
C4	0.0254 (9)	0.0188 (8)	0.0481 (13)	−0.0023 (7)	0.0104 (9)	−0.0041 (8)
C5	0.0308 (10)	0.0330 (10)	0.0244 (9)	0.0088 (8)	−0.0042 (8)	−0.0088 (8)
C3	0.0387 (12)	0.0343 (11)	0.0269 (10)	0.0160 (9)	0.0150 (9)	0.0104 (8)
C6	0.0264 (9)	0.0196 (8)	0.0406 (11)	−0.0068 (7)	0.0110 (8)	−0.0071 (8)
O6	0.0195 (6)	0.0186 (6)	0.0308 (7)	0.0076 (5)	−0.0062 (5)	−0.0057 (5)
C10	0.0244 (9)	0.0344 (11)	0.0411 (12)	−0.0056 (8)	−0.0063 (9)	0.0204 (9)

### Geometric parameters (Å, º)

**Table:**

Sn1—C1	2.124 (2)	C9—C8	1.511 (2)
Sn1—C1^i^	2.124 (2)	C9—H9A	0.9900
Sn1—C2	2.133 (3)	C9—H9B	0.9900
Sn1—O1^i^	2.2665 (12)	C2—H2A	0.9800
Sn1—O1	2.2665 (12)	C2—H2B	0.9800
Sn3—C6	2.118 (2)	C2—H2C	0.9800
Sn3—C10	2.119 (2)	C1—H1A	0.9800
Sn3—C7	2.125 (2)	C1—H1B	0.9800
Sn3—O3	2.1522 (12)	C1—H1C	0.9800
Sn3—O5^ii^	2.4335 (12)	C4—H4A	0.9800
Sn2—C3	2.112 (2)	C4—H4B	0.9800
Sn2—C4	2.120 (2)	C4—H4C	0.9800
Sn2—C5	2.123 (2)	C5—H5A	0.9800
Sn2—O2	2.1587 (12)	C5—H5B	0.9800
Sn2—O6	2.3756 (13)	C5—H5C	0.9800
P1—O3	1.5156 (12)	C3—H3A	0.9800
P1—O2	1.5180 (13)	C3—H3B	0.9800
P1—O1	1.5310 (12)	C3—H3C	0.9800
P1—C9	1.8348 (16)	C6—H6A	0.9800
O5—C8	1.2571 (19)	C6—H6B	0.9800
O5—Sn3^iii^	2.4335 (12)	C6—H6C	0.9800
O4—C8	1.2752 (19)	O6—H1O	0.8900
O4—H1O4	0.8500	O6—H2O	0.8500
C7—H7A	0.9800	C10—H10A	0.9800
C7—H7B	0.9800	C10—H10B	0.9800
C7—H7C	0.9800	C10—H10C	0.9800
			
C1—Sn1—C1^i^	115.13 (11)	P1—C9—H9B	109.0
C1—Sn1—C2	122.43 (6)	H9A—C9—H9B	107.8
C1^i^—Sn1—C2	122.43 (6)	Sn1—C2—H2A	109.5
C1—Sn1—O1^i^	88.44 (6)	Sn1—C2—H2B	109.5
C1^i^—Sn1—O1^i^	89.87 (6)	H2A—C2—H2B	109.5
C2—Sn1—O1^i^	91.58 (3)	Sn1—C2—H2C	109.5
C1—Sn1—O1	89.87 (6)	H2A—C2—H2C	109.5
C1^i^—Sn1—O1	88.44 (6)	H2B—C2—H2C	109.5
C2—Sn1—O1	91.58 (3)	O5—C8—O4	120.75 (14)
O1^i^—Sn1—O1	176.84 (6)	O5—C8—C9	120.30 (14)
C6—Sn3—C10	118.46 (10)	O4—C8—C9	118.94 (14)
C6—Sn3—C7	124.85 (9)	Sn1—C1—H1A	109.5
C10—Sn3—C7	115.59 (10)	Sn1—C1—H1B	109.5
C6—Sn3—O3	90.27 (6)	H1A—C1—H1B	109.5
C10—Sn3—O3	96.87 (7)	Sn1—C1—H1C	109.5
C7—Sn3—O3	93.58 (7)	H1A—C1—H1C	109.5
C6—Sn3—O5^ii^	86.24 (6)	H1B—C1—H1C	109.5
C10—Sn3—O5^ii^	84.57 (6)	Sn2—C4—H4A	109.5
C7—Sn3—O5^ii^	88.65 (7)	Sn2—C4—H4B	109.5
O3—Sn3—O5^ii^	176.51 (5)	H4A—C4—H4B	109.5
C3—Sn2—C4	122.21 (10)	Sn2—C4—H4C	109.5
C3—Sn2—C5	118.33 (10)	H4A—C4—H4C	109.5
C4—Sn2—C5	117.86 (10)	H4B—C4—H4C	109.5
C3—Sn2—O2	92.16 (7)	Sn2—C5—H5A	109.5
C4—Sn2—O2	95.72 (7)	Sn2—C5—H5B	109.5
C5—Sn2—O2	94.76 (7)	H5A—C5—H5B	109.5
C3—Sn2—O6	84.82 (7)	Sn2—C5—H5C	109.5
C4—Sn2—O6	86.99 (7)	H5A—C5—H5C	109.5
C5—Sn2—O6	85.56 (6)	H5B—C5—H5C	109.5
O2—Sn2—O6	176.72 (5)	Sn2—C3—H3A	109.5
O3—P1—O2	112.62 (7)	Sn2—C3—H3B	109.5
O3—P1—O1	112.74 (7)	H3A—C3—H3B	109.5
O2—P1—O1	111.98 (7)	Sn2—C3—H3C	109.5
O3—P1—C9	105.92 (7)	H3A—C3—H3C	109.5
O2—P1—C9	106.33 (7)	H3B—C3—H3C	109.5
O1—P1—C9	106.64 (7)	Sn3—C6—H6A	109.5
C8—O5—Sn3^iii^	120.20 (10)	Sn3—C6—H6B	109.5
C8—O4—H1O4	114.10	H6A—C6—H6B	109.5
P1—O1—Sn1	133.13 (7)	Sn3—C6—H6C	109.5
P1—O3—Sn3	147.67 (8)	H6A—C6—H6C	109.5
Sn3—C7—H7A	109.5	H6B—C6—H6C	109.5
Sn3—C7—H7B	109.5	Sn2—O6—H1O	117.00
H7A—C7—H7B	109.5	Sn2—O6—H2O	123.00
Sn3—C7—H7C	109.5	H1O—O6—H2O	104.00
H7A—C7—H7C	109.5	Sn3—C10—H10A	109.5
H7B—C7—H7C	109.5	Sn3—C10—H10B	109.5
P1—O2—Sn2	147.66 (8)	H10A—C10—H10B	109.5
C8—C9—P1	112.86 (11)	Sn3—C10—H10C	109.5
C8—C9—H9A	109.0	H10A—C10—H10C	109.5
P1—C9—H9A	109.0	H10B—C10—H10C	109.5
C8—C9—H9B	109.0		
			
O3—P1—O1—Sn1	91.91 (11)	O1—P1—O2—Sn2	121.26 (15)
O2—P1—O1—Sn1	−36.34 (12)	C9—P1—O2—Sn2	−122.63 (15)
C9—P1—O1—Sn1	−152.26 (9)	C3—Sn2—O2—P1	−130.11 (17)
C1—Sn1—O1—P1	76.70 (11)	C4—Sn2—O2—P1	−7.43 (17)
C1^i^—Sn1—O1—P1	−168.15 (11)	C5—Sn2—O2—P1	111.23 (16)
C2—Sn1—O1—P1	−45.74 (10)	O3—P1—C9—C8	−151.17 (11)
O2—P1—O3—Sn3	164.46 (14)	O2—P1—C9—C8	−31.14 (13)
O1—P1—O3—Sn3	36.55 (17)	O1—P1—C9—C8	88.51 (12)
C9—P1—O3—Sn3	−79.71 (16)	Sn3^iii^—O5—C8—O4	−12.4 (2)
C6—Sn3—O3—P1	−142.37 (16)	Sn3^iii^—O5—C8—C9	166.22 (11)
C10—Sn3—O3—P1	−23.64 (17)	P1—C9—C8—O5	−80.06 (17)
C7—Sn3—O3—P1	92.69 (16)	P1—C9—C8—O4	98.60 (16)
O3—P1—O2—Sn2	−7.05 (18)		

Symmetry codes: (i) −*x*+1, *y*, −*z*+1/2; (ii) *x*+1/2, *y*+1/2, *z*; (iii) *x*−1/2, *y*−1/2, *z*.

### Hydrogen-bond geometry (Å, º)

**Table:**

*D*—H···*A*	*D*—H	H···*A*	*D*···*A*	*D*—H···*A*
O6—H1*O*···O5^iv^	0.89	1.83	2.693 (2)	164
O6—H2*O*···O1^iv^	0.85	1.88	2.706 (2)	161
C9—H9*A*···O4^v^	0.99	2.51	3.227 (2)	129

Symmetry codes: (iv) *x*−1/2, *y*+1/2, *z*; (v) −*x*+1/2, −*y*+3/2, −*z*.
